# Brain mapping during resection of high-grade brain arteriovenous malformation

**DOI:** 10.3171/2024.10.FOCVID24107

**Published:** 2025-01-01

**Authors:** Ari D. Kappel, Mitali Bose, Matthew Toczylowski, Nirav J. Patel

**Affiliations:** 1 Department of Neurosurgery, Brigham and Women’s Hospital, Harvard Medical School, Boston, Massachusetts; and; 2 SpecialtyCare, Intraoperative Neuromonitoring, Boston, Massachusetts

**Keywords:** subcortical mapping, cerebrovascular, neurophysiology, arteriovenous malformation, brain AVM, bilateral interhemispheric, hemorrhagic stroke

## Abstract

Eloquent brain creates a challenge when resecting brain arteriovenous malformations (bAVMs). Here the authors present their technique of using subcortical motor mapping as an adjunct to increase safety during resection of a high-grade bAVM involving somatosensory cortex as well as cortical spinal tracts and visual tracts. After a bilateral craniotomy, they use direct cortical stimulation of the left motor cortex and subcortical stimulation using a suction stimulator to dynamically map motor tracts during the resection. They get within 3 mm of the corticospinal tracts by stimulation.

The video can be found here: https://stream.cadmore.media/r10.3171/2024.10.FOCVID24107

## Transcript

Brain mapping during resection of high-grade brain arteriovenous malformation surgery.

### 0:26 Patient Presentation and Preoperative Imaging.

This is a large AVM in the left cerebral cortex which has deep venous drainage and is fed by the ACA and PCA territory. The eloquence is what mapping is really used for here. The cortical spinal tracts are just anterolateral in yellow, and the visual fibers are posterior. The AVM comes into the sensory cortex proper and gets very close to the posterior thalamus. We will use mapping for these eloquent areas. The approach is a semisitting supine biparietal craniotomy to get the ACA/PCA territory arteries first without embolization^1,2^ and then intraoperative angiograms as necessary.

### 1:14 Surgical Procedure.

Here is our neurophysiologist adding the motor and sensory leads into the scalp and then posteriorly the visually evoked leads.^3,4^ She’s now putting visually evoked glasses on the patient’s eyes and securing it with tape. The bicoronal incision is opened, and we have access to the skull with burr holes placed to make two separate craniotomies, one on the right and then dissect the sagittal sinus from the one on the left, which is safest. The convexity dura are brought together in the midline and sutured in order to avoid any more bleeding. We begin placing the lead on the left motor cortex and checking that we’re on motor, and you can see the visual evoked potential grid on the occipital lobe.^5,6^

### 2:07 AVM Resection: ACA/PCA Targets and Motor Mapping.

Then going in the deep interhemispheric fissure to find the ACA target with the Brainlab overlay in the microscope, this is facilitated. We find the ACA, we place a temporary clip on that, and now I’m opening the falx to get from the right to the left. This facilitates the medial exposure of the AVM where the PCA artery is going to be found against the vein of Galen. Navigation, again, giving confidence to the dissection. The clip is placed on the PCA, and we go to the anterior aspect of the AVM against the splenium now to begin dissection toward the lateral ventricle, with the goal being to get the posterior lateral choroidal closing as much of the inflow as possible for this large AVM before a real resection occurs. Now, on the margin of the AVM nidus, the bipolar and suction are utilized down the margin. Here we use the stimulator to assure ourselves of where motor is on the medial aspect before making any cuts into the cortex to go along the superior lateral aspect of the AVM. We find sensory and go just behind that, making a cortisectomy along the posterior cingulate.

### 3:49 AVM Resection Continued: Lateral Ventricle and Posterior Lateral Choroidal.

The navigation aids in the trajectory of this dissection, as I want to stay right on the nidus, in order to get down to the ventricle in an efficient manner. Any of the loops of the AVM should be brought back into the nidus. Because this is a rather diffuse AVM, it does pose a challenge as to where the border is, but we continue dissecting, progressing in the white matter. Remember, the dissection is really just the bipolar suction. And here we see the lateral ventricle. This is a 0.5-mm bipolar. And the goal being to find that posterior lateral choroidal artery. This is the big draining vein in the ventricle itself. And here we get around it, and we start to see the artery coming in. The posterior lateral choroidal. It’s coagulated. And a clip is placed on it, both to mark it and to reassure ourselves that it is not going to bleed. Then it is cut. This has decreased the total blood flow to this AVM.

### 5:27 AVM Resection: CST, Thalamus, and Subcortical Mapping.

Here the picture-in-picture confirms our position. The next step is to go anterolateral where the corticospinal tracts as well as the thalamus could be found. We’re using the subcortical mapping, and at that, my neurophysiologist tells me I am now 1 cm away from the leg and arm, as this picture demonstrates, as does the navigation. Then I turn my attention posteriorly to the remainder of the dissection. Now, what was challenging here was that the AVM did come out in piecemeal fashion, certainly not something we want to do routinely, but the blood supply had been cut and it was diffuse and that was how it came out. Here the main draining vein in the ventricle is cut. Now this dissection certainly took a long time, but for the purposes of this video illustrating the brain mapping, much of that has been left out. We remain in the ventricle, and pieces of the nidus come out posteriorly. Now the anterior margin was difficult; therefore, at this point I elected to stop and get an angiogram. You can see either side of the falx. The motor strip is removed.

### 7:17 AVM Resection: Cerebral Angiogram, Resection Day 2, and Subcortical Mapping.

We do a soft closure and do an angiogram. And there is a small residual early vein, and we come back on a 2nd day, coming slightly more anterior along the corpus callosum, cross-clamping the artery more approximately. Now we’re going to be even closer to cortical spinal, so the suction stimulator is very, very helpful. Subcortical mapping showed that we got very close to leg at 3 mA, which translates to 3 mm, giving the surgeon confidence.^7^ I try to stay right on the nidus, out of the corticospinal tracts, small movements guided by both the resection of what I see under the microscope, as well as the feedback of the subcortical mapping. And that’s a constant communication between the surgeon and the neurophysiologist. The goal in my mind is to stay right on the AVM, and that means to follow those nidal vessels both medially and laterally. We focus both on the lateral aspect and now the medial aspect in the ventricle. And it’s important not to go across the ventricle because the posterior thalamus is just adjacent to that tissue there. And you can see there was that nidus that was demonstrated in the angio. That’s an en passant artery and is left intact.

### 9:08 Video Fluoroscopy, Cerebral Angiogram, Final Resection, and Closure.

ICG demonstrates better flow, less early venous drainage. I do another angiogram but continue to find an early vein, a different one than the last angiogram. So, we continue our resection, very focused along the superior posterior margin of the dissection, the cingulate gyrus. At this point, we can be a little more aggressive about the distance from the nidus as we are past motor, adjacent to sensory, and the ventricle is again exposed and EVD catheter is placed. And the remainder of the AVM is then taken out and we prepare for a final angiogram, where there was no early venous drainage. At this point, we do our final closure where the dura is brought back together in the midline, craniotomy is put back, a JP drain, and then really good galeal stitches are used to hold this bicoronal together.

### 10:26 Postoperative Course.

Postoperative she had a cranioplasty and VP shunt for hydrocephalus and at 2-year follow-up has a modified Rankin score of 1 for numbness and a small superior quadrant field cut, but overall returned to her life and is doing very well.

## Disclosures

The authors report no conflict of interest concerning the materials or methods used in this study or the findings specified in this publication.

## Author Contributions

Primary surgeon: Patel. Assistant surgeon: Kappel. Editing and drafting the video and abstract: Kappel, Toczylowski. Critically revising the work: Kappel, Bose. Reviewed submitted version of the work: Kappel, Bose. Approved the final version of the work on behalf of all authors: Patel. Surgical neurophysiology specialist: Toczylowski.

## Supplemental Information

### Patient Informed Consent

The necessary patient informed consent was obtained in this study.
